# In praise of conscious awareness: a new framework for the investigation of “continuous improvement” in expert athletes

**DOI:** 10.3389/fpsyg.2014.00769

**Published:** 2014-07-16

**Authors:** John Toner, Aidan Moran

**Affiliations:** ^1^School of Sport, Health and Exercise Sciences, University of HullHull, UK; ^2^School of Psychology, University College DublinDublin, Ireland

**Keywords:** expertise, continuous improvement, attention, embodiment, bodily awareness

## Abstract

A key postulate of traditional theories of motor skill-learning (e.g., Fitts and Posner, 1967; Shiffrin and Schneider, 1977) is that expert performance is largely automatic in nature and tends to deteriorate when the performer “reinvests” in, or attempts to exert conscious control over, proceduralized movements (Masters and Maxwell, 2008). This postulate is challenged, however, by recent empirical evidence (e.g., Nyberg, in press; Geeves et al., 2014) which shows that conscious cognitive activity plays a key role in facilitating further improvement amongst expert sports performers and musicians – people who have *already* achieved elite status (Toner and Moran, in press). This evidence suggests that expert performers in motor domains (e.g., sport, music) can strategically deploy conscious attention to alternate between different modes of bodily awareness (reflective and pre-reflective) during performance. Extrapolating from this phenomenon, the current paper considers how a novel theoretical approach (adapted from Sutton et al., 2011) could help researchers to elucidate some of the cognitive mechanisms mediating continuous improvement amongst expert performers.

Many traditional and contemporary theories of motor learning emphasize the apparently “spontaneous” nature of skilled performance. For example, Fitts and Posner (1967) and Shiffrin and Schneider (1977) argued that skilful action runs “automatically” or “procedurally.” Similarly, Dreyfus (2002) claimed that expert performance proceeds “without calculating and comparing alternatives … what must be done, simply is done” (p. 372). Common to these accounts of expertise is the belief that any form of conscious involvement during on-line skill execution is likely to prove deleterious to movement and performance proficiency (Beilock et al., 2002). Challenging these dominant perspectives on skill-learning, however, is an emerging body of theory (e.g., Breivik, 2013; Toner and Moran, in press; Winter et al., 2014) and empirical evidence (see Nyberg, in press) which suggests that “continuous improvement” (i.e., the phenomenon whereby certain skilled performers appear to be capable of increasing their proficiency even though they are already experts) at the elite level of sport is heavily reliant upon the performer’s ability to move efficiently between reflective and unreflective modes of bodily awareness. For example, experts are often required to pay conscious attention to, or “reinvest” in the training context, when their movements become “attenuated” (see Collins et al., 1999) because they believe that in order to optimize their performance they must “experiment with and research their moving body” (Ravn and Christensen, in press).

Interestingly, evidence suggests that many skilled performers remain “somaesthetically” aware (i.e., focusing on the proprioceptive “feel”, Shusterman, 2008) of their movement during on-line skill execution (in the performance context; see Nyberg, in press) and can use global or holistic cue words to improve performance proficiency under pressurized conditions (see Mullen and Hardy, 2010). Therefore, instead of relying wholly on unthinking spontaneity to guide their performance, elite athletes appear to alternate between different modes of cognitive processing, and also between types of bodily self-awareness (i.e., reflective and pre-reflective) in practice and performance contexts. Here athletes might adopt what Colombetti (2011) refers to as a *reflective* awareness of their bodily selves when they consider their intentions or actions and assess whether they are appropriate to a certain situation. By contrast, *pre-reflective* awareness occurs when we are immersed in an activity but our attention is not on our bodily selves. However, in the latter case, Colombetti (2011) argues that the body is not entirely invisible or absent from experience as it remains “as a source of feeling, affect, agency and expressivity” (p. 27).

In a recent paper (see Toner and Moran, in press), we argued that self-focused attention (including reflective bodily awareness) plays an important role in allowing skilled athletes to refine inefficient movements during deliberate practice. The present paper extends this argument by postulating that skilled performers are capable of strategically allocating attention, and hence alternating between reflective and pre-reflective modes of awareness, in order to meet the requirements of dynamically unfolding and contextually contingent performance environments. Furthermore, we argue that influential theoretical accounts (such as self-focus theories; e.g., Beilock and Carr, 2001) used by researchers to identify the cognitive mechanisms underpinning performance at the expert level may be unable to adequately capture or explain the dynamical (i.e., that it may be freely allocated) nature of attentional processing amongst elite performers. Arising from these arguments, we propose that Sutton et al.’s (2011) “Applying intelligence to the reflexes” (AIR) approach, and a number of methodologies which aim to uncover participants’ phenomenological descriptions of training and performance, may be better suited in achieving this latter aim.

Over the last decade or so, experimental psychologists have used standardized laboratory tasks in order to identify the attentional processes that govern skilled movement and performance in sport (e.g., Beilock and Carr, 2001; Jackson et al., 2006). For example, Beilock et al. (2002) found that when skilled golfers attended to a specific aspect of their putting technique (e.g., the exact moment that the clubhead finished its follow-through), their performance was impaired relative to counterparts in a dual-task condition who putted while performing a secondary task (an auditory tone-monitoring activity). Further evidence for the detrimental effects of skill-focused attention on skilled performance have been found in a baseball batting task (Gray, 2004); in soccer dribbling (Ford et al., 2005); and in a golf putting task (Mullen and Hardy, 2000). According to self-focus theories of attention (including Beilock and Carr, 2001, “explicit monitoring hypothesis” and Masters, 1992, “conscious processing hypothesis”) attending to the step-by-step component processes of a proceduralized skill results in its control structures being broken down into a sequence of smaller, separate, independent units. Ultimately, this creates the opportunity for error that was not present in the “chunked” control structure (Beilock et al., 2002). Accordingly, performers have been advised to avoid focusing on their bodily movement and, instead, to “shift the focus to the external world, in particular on the impact or effect of one’s behaviors” (Weiss and Reber, 2012, p. 174).

At first glance, it would seem that the case is quite clear – any form of conscious processing that directs the performer’s attention to their bodily movement is likely to disrupt fluent skill execution. However, a closer look at the laboratory-based evidence base would suggest that its findings must be interpreted with caution. To explain, in each of the aforementioned studies athletes were asked to focus on a feature of their movement which they may never have previously focused on (and hence never practiced doing so). Indeed, few studies have sought to capture athletes’ attentional processes over time (e.g., between the “off-season” and competitive season) or across different situations (e.g., when recovering from injury). Given the dearth of studies in psychology on the temporal and/or contextual dynamics of attentional processes, we question the degree to which available evidence supports the received wisdom that conscious attention will *inevitably* disrupt skilful performance. In fact, recent research has begun to cast doubt upon the validity of this latter assumption. For example, Nyberg (in press) found that elite freeskiers attended to on-line skill execution in order to identify any features of their movement which might require alteration/adjustment in order to maintain performance proficiency. In addition, a large volume of evidence indicates that elite performers are capable of flexibly allocating their attention (i.e., moving from reflective to pre-reflective modes of bodily awareness) dependent upon the context-specific demands confronting them during training and competitive performance (see Bernier et al., 2011) or the challenges (e.g., injuries, slumps) that they will inevitably face at some stage during their careers (see Collins et al., 1999). In stark contrast to self-focus theories, these emergent findings suggest that skilled performance is likely to be impeded if the “proceduralization” of skills is excessive (see Ericsson, 2006) – because experts must be able to deliberately access and strategically re-route any semi-automated routines in order to facilitate “continuous improvement” (Montero, 2010; Breivik, 2013).

Against this background, we argue that there is ample empirical evidence that “continuous improvement” at the elite level is heavily dependent upon the performer’s ability to effectively utilize reflective modes of bodily awareness. Skill-focused attention (including conscious bodily awareness) appears to be a key feature of skilled performance because athletes operating at this level are driven by the desire to learn “new and better techniques” (Breivik, 2007, p. 127). For example, despite having won eight medals at the Beijing Olympics, Michael Phelps decided to change his freestyle technique in a bid to increase his sprinting speed (Andersson, 2009). Moreover, athletes will inevitably experience injury, fatigue, growth and aging which may disrupt habitual movement (see Bissell, 2013; Eden, 2013) and require them to correct, relearn and adjust their spontaneous performance (Shusterman, 2008). In fact, research on the topic of “skill recovery” or “skill refinement” shows that skilled athletes who are attempting to regain prior levels of performance often deliberately reinvest conscious control to restore or refine habitual movements in sports such as javelin throwing and swimming (Hanin et al., 2004). In these studies, researchers have helped athletes regain or refine disrupted movement patterns by encouraging them to become more consciously aware of the somaesthetic differences between current (problematic) and desired actions.

Somaesthetic awareness appears to play an important role in “continuous improvement” by allowing performers to identify movements that are causing them discomfort or outcomes which are unusual or undesirable. Indeed, evidence suggests that these forms of self- awareness are important mediators of “flow” or optimal competitive performance in sport. On the basis of their pioneering research on flow in sport, Jackson and Csikszentmihalyi (1999) argued that “without self-awareness an athlete misses important cues that can lead to a positive change in performance” (p. 105). According to these authors, self-awareness simply means paying attention to cues provided by movements, and making adjustments to your actions when something is amiss. Athletes may use reflective bodily awareness to identify “attenuated” habits in the performance context and subsequently adjust problematic movements in the training context. However, evidence suggests that performers may also choose to adjust problematic movements during on-line execution during competition. To illustrate, Collins et al. (2001) found that elite weightlifters chose to consciously modify their movement during competition in order to maintain movement proficiency. Similarly, Nyberg found that elite freeskiers learn how to discern (i.e., through “focal awareness”) their rotational velocity to such an extent that they “know whether they will be able to perform the trick the way it was intended without adjustments, or whether they will need to make adjustments during the flight phase” (p. 7). In this study, elite free skiers were video recorded during practice and subsequently interviewed using a technique known as stimulated recall (SR – a method for enhancing reflection by recalling situations through audiotapes or video recordings). Nyberg suggests that these performers rely on their focal awareness (which is conscious and might include knowledge of their velocity and how they need to modify it) and their subsidiary awareness which is “less conscious” and includes knowledge of the “particulars” such as the friction of the snow and their feelings of previous jumps. These elite performers were found to navigate their focal awareness by rapidly shifting its target even in the midst of the activity itself. For one performer, this meant that he was focally and embodiedly aware of his rotational velocity while in the air but could quickly change his awareness to take into account environmental conditions such as his position in relation to the targeted landing area. Clearly, these findings suggest that some performers seek to counteract automaticity by ensuring that certain features of performance are subject to strategic control.

Although some performers may choose to reinvest conscious attention by adjusting movement in the performance context, others may choose to use cue words as “instructional nudges” (i.e., explicit verbal phrases or maxims: see Sudnow, 2001; Sutton, 2007) in order to “re-route” embodied routines. According to Sutton et al. (2011) cues may allow the performer to build and access “flexible links between knowing and doing” (p. 95). Cue words appear to represent forms of thinking and remembering which can, in some circumstances, allow performers to animate the kinesthetic mechanisms of skilled performance. To illustrate, Jenkins (2007) interviewed 113 European tour golfers and found that 70% of these performers used at least one “swing thought” (i.e., a form of cue word) during on-line skill execution. Clearly, certain forms of mindedness or conscious processing are a common feature of elite competitive performance.

The preceding evidence would suggest that a better understanding of the cognitive processes mediating continuous improvement at the elite level of skilled performance can be achieved only by adopting a theoretical framework which can account for the dynamic nature of attentional processing. Therefore, instead of explanations (e.g., self-focus theories) that emphasize the proceduralized nature of skilled performance, we may require theoretical accounts which focus on the *interchanging* phases or stages of learning (Shusterman, 2008, 2009) that appear to characterize training and performance at the elite level of sport. Consequently, we propose that Sutton et al.’s (2011) AIR approach may help researchers to explain how performers can alternate between different modes of processing. Briefly, Sutton et al.’s (2011) model is cyclical in the sense that the maintenance and enhancement of performance efficiency requires the “rapid switching of modes and styles” (2011, p. 93) within the training and performance context. This framework proposes that expert skill relies on a mindedness that “facilitates the dynamic flexibility of attention, allowing it to be allocated freely and in a way that best meets contingent contextual demands” (Geeves et al., 2014, p. 676). Accordingly, Geeves et al. (2014) argue that expert performers may determine the amount of attention they need to pay to certain processes in the practice context (depending on their current level of performance) and during on-line performance (according to the situational demands presented to them).

Additionally, we propose that the AIR model may help researchers to interpret the accumulating body of empirical evidence which suggests that skilled performers seek to avoid automaticity by ensuring that performance remains open to strategic control in both the practice and performance context. Sutton et al. (2011) argued that there are a number of different ways in which embodied coping is minded or mindful (varying across individuals, task domains, and cultures) and recommend that we search for forms of theorizing that highlight these differences by exploring what actually happens to performers as they “direct attention to kinesthetic cues in increasingly skilful ways” (p. 96). Given the preceding evidence documenting the mindful nature of expertise, it would also seem important to identify methodological approaches that will help researchers capture the attentional switching mechanisms that appear to underpin “continuous improvement” at the elite level of sport. Specifically, in order to understand embodied perspectives of experience researchers may wish to adopt methodological approaches that are “truly grounded in the carnal realities of the lived sporting bodies” (Hockey and Allen-Collinson, 2007, p. 116). Some researchers have recently taken up this challenge by drawing on participants’ phenomenological insights to better understand how embodied expertise is shaped by training and performance. For example, Ravn and Christensen (in press) utilized a phenomenology-related analysis of qualitative data (including participant observations and interviews) with an elite golfer to explore how the “described experience comes into being rather than what this experience means to the subject” (p. 5). The principal author observed Line (an elite golfer), between 5 and 8 h each day, over 5 days of training. The researchers drew on notes taken during these observations to invite Line to describe her practice sessions and experiences in detail. The researchers subsequently analyzed the data by looking for “petite generalizations” (i.e., generalizations that regularly occurred in the case study) relating to how Line used her awareness of bodily sensations during training. Overall, the findings suggested that training at the elite level is not just about handling the physicality of the body but also about listening to it and regulating how it should “feel” in order to perform optimally (see Nyberg, in press, for a similar methodological approach).

In another study, Bernier et al. (2011) used a naturalistic investigation to explore the attentional foci adopted by elite golfers in training and performance contexts. The initial phase of the study involved filming participants (for 60 min) in a training session during the winter (non-competitive) season. The second phase took place during the competitive season and involved filming participants over the course of a complete round (i.e., 18 holes of play) in a professional competition. Self-confrontation interviews, based on video footage, were conducted within 2 h of the completion of the training session and competitive performance. The interviews sought to encourage SR by asking each participant to view the videotape with the researcher and to recall and describe what thoughts he was processing during the training session or competition. Initially, the participants and the researcher watched the first situations on the video recording (i.e., the first training exercise and the first three holes of the competition). Having discussed the attentional foci adopted during these situations, the participant was asked to indicate specific circumstances that he considered relevant to analyse. These situations included specific exercises during the training sessions or great/poorly executed strokes during competition. Each sequence involved an action (e.g., a practice drill or a shot), the preparatory phase (e.g., the pre-shot routine), and the step following this action (e.g., walking to the next shot). Participants were urged to express their thoughts during each sequence, rather than being asked to explain “their solution for the task or to provide a summary of the general strategy adopted” (Bernier et al., 2011, p. 331). Inductive content analysis revealed that these elite golfers adapted their attentional focus depending on the context. That is, golfers were found to flexibly adjust their attentional focus (moving back-and-forth between internal and external foci) across the preparatory, execution and evaluative stages of training and competitive performance.

In summary, a significant volume of evidence shows that skilled performers’ foci of attention may change dramatically over the course of a competitive season (e.g., to deal with “attenuated” movement patterns) or during a competitive event (i.e., between preparation, execution, and evaluation). Unfortunately, as most studies of attentional processes in psychology are limited to static snapshots of the phenomena of interest, they shed little light on the dynamical nature of attention. Against this background, the current paper has argued how Sutton et al.’s (2011) AIR approach may provide researchers with a more detailed understanding of the embodied nature of skilful action and performance – thereby helping to explain how athletes strategically allocate attentional resources in seeking to maintain and enhance performance proficiency. In order to shed light on the complex and dynamic attentional mechanisms that mediate “continuous improvement” in expert performers researchers may need to use a variety of methods including both standardized laboratory techniques (e.g., occlusion paradigms, eye-tracking) and phenomenological and naturalistic investigations (e.g., SR). Together, these approaches may help researchers understand how and why performers alternate between reflective and pre-reflective modes of bodily awareness across training and performance contexts.

## Conflict of Interest Statement

The authors declare that the research was conducted in the absence of any commercial or financial relationships that could be construed as a potential conflict of interest.
